# Ultrasensitive detection of a responsive fluorescent thymidine analogue in DNA *via* pulse-shaped two-photon excitation†

**DOI:** 10.1039/d4cp03391d

**Published:** 2024-10-08

**Authors:** Alexandra E. Bailie, Henry G. Sansom, Rachel S. Fisher, Ryo Watabe, Yitzhak Tor, Anita C. Jones, Steven W. Magennis

**Affiliations:** a EaStCHEM School of Chemistry, The University of Edinburgh West Mains Road Edinburgh EH9 3JJ UK a.c.jones@ed.ac.uk; b School of Chemistry, University of Glasgow, Joseph Black Building, University Avenue Glasgow G12 8QQ UK steven.magennis@glasgow.ac.uk; c Department of Chemistry and Biochemistry, University of California, San Diego 9500 Gilman Drive La Jolla CA 92093 USA

## Abstract

Fluorescent base analogues (FBAs) are versatile nucleic acid labels that can replace a native nucleobase, while maintaining base pairing and secondary structure. Following the recent demonstration that free FBAs can be detected at the single-molecule level, the next goal is to achieve this level of detection sensitivity in oligonucleotides. Due to the short-wavelength absorption of most FBAs, multiphoton microscopy has emerged as a promising approach to single-molecule detection. We report the multiphoton-induced fluorescence of 5-(5-(4-methoxyphenyl)thiophen-2-yl)-6-aza-uridine (MeO^th^aU), a polarity-sensitive fluorescent thymidine analogue, as a nucleoside, and in two single-stranded deoxyribo-oligonucleotides, with and without their complementary strands. Ensemble steady-state and time-resolved measurements in dioxane, following one-photon and two-photon excitation, reveals both strongly and weakly emissive species, assigned as rotamers, while in Tris buffer there are additional non-emissive states, which are attributed to tautomeric forms populated in aqueous environments. The two-photon (2P) brightness for MeO^th^aU is highest as the free nucleoside in dioxane (10 GM) and lowest as the free nucleoside in Tris buffer (0.05 GM). The species-averaged 2P brightness values in DNA are higher for the single strands (0.66 and 0.82 GM for sequence context AXA and AXT, respectively, where X is MeO^th^aU) than in the duplex (0.31 and 0.25 GM for AXA and AXT, respectively). Using 2P microscopy with pulse-shaped broadband excitation, we were able to detect single- and double-stranded oligos with a molecular brightness of 0.8–0.9 kHz per molecule. This allowed the detection of as few as 7 DNA molecules in the focus, making it the brightest responsive FBA in an oligonucleotide reported to date.

## Abstract 2

Incorporation of a responsive fluorescent thymidine analogue in oligonucleotides allows detection of as few as seven DNA molecules using two-photon microscopy.

## Graphical abstract

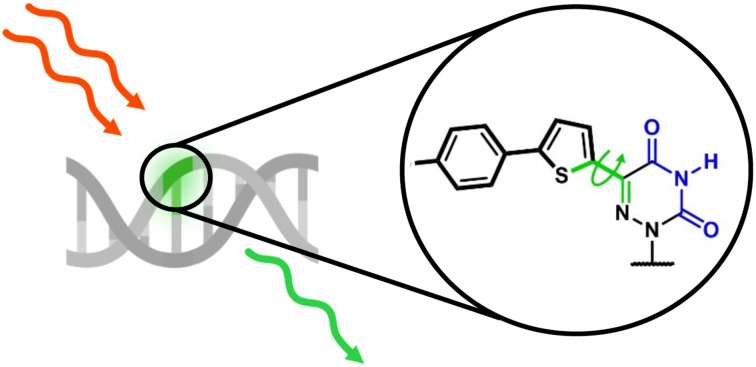

## Introduction

The application of powerful fluorescence-based techniques to the study of DNA requires the introduction of extrinsic fluorophores, since the natural DNA bases have vanishingly small fluorescence quantum yields.^1^ Fluorescent labelling can be achieved with minimal perturbation to the nucleic acid structure through the replacement of a single natural base in an oligonucleotide by a fluorescent base analogue (FBA), a fluorophore with close structural resemblance to the natural bases, including the ability to form Watson–Crick base pairs.^2–4^ The use of responsive FBAs, whose photophysical properties respond to local molecular environment, takes this approach beyond merely making DNA molecules visible for detection and imaging, into the realms of exploring the site-specific conformational properties of DNA and the effects of protein-binding events on those properties.^5^

2-Aminopurine (2AP) is the archetypal responsive FBA; first-reported in 1969^6^ it remains the most widely used FBA and has delivered a wealth of information on the mechanisms of enzymes that modify or repair DNA.^4,5,7^ 2AP is remarkable in the similarity of its structure to that of adenine (6-aminopurine) and the exquisite sensitivity of its fluorescence properties to inter-base stacking interactions. However, 2AP has significant shortcomings, notably the need for UV excitation (around 300 nm) and its low quantum yield when inserted in oligonucleotides. This has stimulated efforts to develop new FBAs that absorb at longer wavelengths and have greater brightness in DNA.^3,4,8^ However, while there has been some success in red-shifting excitation wavelengths, these still lie generally in the UV region, below 400 nm, and, in most cases, quantum yields in oligonucleotides are not significantly higher than for 2AP (a few percent).

In the context of responsive FBAs, thienoguanosine (^th^G) is a notable new isomorphic analogue that retains relatively high brightness in oligos, with a quantum yield of ∼0.15 in duplex DNA, and a well-characterised photophysical response to inter-base interactions.^9–11^ However, although its excitation wavelength is considerably red-shifted relative to 2AP, it remains below 400 nm. Two recently developed FBAs with expanded aromatic systems, pentacyclic adenine (pA)^12^ and ABN,^13^ a tricyclic pyrimidine analogue, show outstanding brightness in oligos and relatively long excitation wavelengths. The quantum yield of pA is as high as 0.22 in duplexes, depending on sequence context, and its absorption spectrum extends just beyond 400 nm, with *λ*_max_ at 390 nm. Although the quantum yield and fluorescence decay parameters of pA are strongly affected by sequence context, a systematic relationship between its photophysical properties and molecular environment has not been established, and it seems unlikely to find application as a responsive probe. ABN shows an exceptionally high quantum yield of 0.5 in duplexes and an unprecedented long-wavelength absorption, with *λ*_max_ at 450 nm; it appears to be unresponsive, its quantum yield and emission wavelength are insensitive to sequence context, but a detailed study of its photophysics in oligonucleotides is yet to be reported.

2AP and other responsive FBAs have proved indispensable in nucleic acid research, particularly in studies of DNA-binding proteins, but the conformational complexity of DNA hinders the deduction of mechanistic detail from ensemble photophysical measurements. Much greater insight into the dynamical properties of DNA and the mechanisms of DNA–enzyme processes could be gained from single-molecule studies at the individual base level, but this cannot be achieved with currently available FBAs. As well as improved brightness, there is a need for longer excitation wavelengths to minimise background fluorescence, but this leads inevitably to structures that no longer resemble a canonical base. To overcome this paradox, an alternative strategy is the use of 2-photon (2P) absorption to enable long-wavelength excitation around 800 nm, while retaining relatively small aromatic systems.^14–19^ Using pulse-shaped, broadband, femtosecond laser excitation we have shown this to be a promising approach, exemplified by the detection of pA in a single-strand oligonucleotide at the level of 5 molecules,^20^ and the single-molecule detection of ABN as the free nucleoside.^13^

**Fig. 1:**
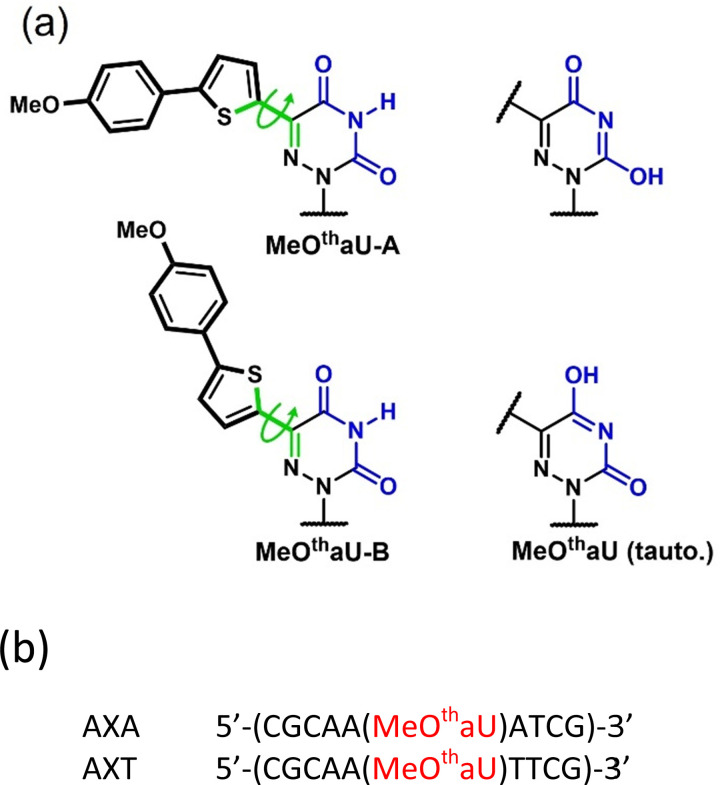
(a) Structures of the two rotamers of MeO^th^aU and the two enol tautomers of the aza-uridine core. (b) Nomenclature and sequences of MeO^th^aU-containing oligonucleotides. Sequences are named according to the bases neighbouring MeO^th^aU on the 5′- and 3′-sides, respectively, with X denoting MeO^th^aU. Duplexes were formed by hybridization with the complementary strand as described in the Experimental section.

In our quest for ultrasensitive detection of a responsive FBA, we have been investigating multiphoton excitation of a series of extended 6-aza-uridine ribonucleosides developed by Tor and coworkers.^21^ In these thymidine analogues, a thiophene is conjugated at the 5-position of 6-aza-uridine and the aromatic system is further extended by attaching an electron-donating phenyl group to the thiophene, as exemplified in Fig. 1. Members of the series with electron-rich substituents (methoxy, hydroxy or dimethylamino) show pronounced sensitivity of quantum yield to solvent polarity, increasing by more than an order of magnitude on going from water to dioxane.^21^ As might be expected, we found that the 2P cross-section increased with increasing electron-donating capability of the substituent, as shown in Table S1 (ESI†),^22^ leading to an exceptionally high value (for an FBA) of 90.0 GM for the dimethylamino-substituted analogue, DMA^th^aU. As reported previously, using multiphoton excitation, we were able to detect DMA^th^aU, at the single-molecule level, as the free nucleoside in aqueous solution.^23^ Given that its quantum yield increases greatly in non-polar solution, we were encouraged to pursue its single-molecule detection in oligonucleotides. Unfortunately, DMA^th^aU proved challenging to incorporate into oligonucleotides. However, we were able to synthesise oligonucleotides containing the methoxy analogue, MeO^th^aU (Fig. 1). Although the 2P cross-section of MeO^th^aU, 13 GM, is much less than that of DMA^th^aU, its 2P-brightness in dioxane remains promising (due to its significantly higher quantum yield), 10 GM compared with 18 GM for DMA^th^aU (Table S1, ESI†). This also compares favourably with 2P-brightness values for other unusually bright FBAs: 5.8 GM for pA and 2.8 GM for 2CNqA (an unresponsive, quadracyclic adenine analogue).^24^

In ensemble fluorescence measurements, we have examined the responsive photophysics of MeO^th^aU when incorporated in single- and double-strand oligonucleotides (sequences shown in Fig. 1), under one- and two-photon excitation. Time-resolved fluorescence measurements reveal that, within the oligos, MeO^th^aU exists as several ground-state species, some of which are highly emissive and some non-emissive. Using fluorescence correlation spectroscopy (FCS) with pulse-shaped two-photon excitation, we have shown that the high brightness of the emitting states can be exploited to enable detection of MeO^th^aU-containing duplex oligonucleotides at the level of as few as seven molecules, suggesting that single-molecule studies of DNA structure and dynamics using FBAs is within reach.

## Results and discussion

The synthesis and characterization of the MeO^th^aU nucleoside has been reported previously.^21^ Details of the synthesis of oligonucleotides and their characterisation by LC-ESI-MS is provided in the ESI† (Fig. S1–S4).

### 1P photophysics

#### Free MeO^th^aU nucleoside

The photophysical properties of the nucleoside were measured in two solvents, dioxane and Tris buffer, to further explore the previously reported strong influence of environmental polarity^21^ and assist in interpreting the effect of incorporation in oligonucleotides.

**Fig. 2:**
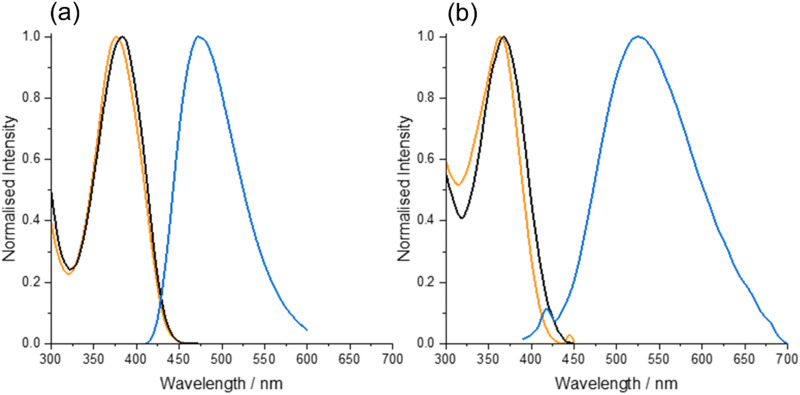
(a) Normalised absorption spectrum (black), excitation spectrum (orange) at emission wavelength 470 nm, and emission spectrum (blue) at excitation wavelength 380 nm, for MeO^th^aU in dioxane. (b) Normalised absorption spectrum (black), excitation spectrum (orange) at emission wavelength 525 nm, and emission spectrum (blue) at excitation wavelength 375 nm, for MeO^th^aU in Tris buffer. (Weak water-Raman bands are evident on the excitation and emission profiles in Tris, due to the low fluorescence quantum yield.)

The absorption, excitation and emission spectra of the free nucleoside in the two solvents are shown in Fig. 2.

In both solvents, there is imperfect overlap of the excitation spectrum and the absorption spectrum, indicating a variation of fluorescence quantum yield with absorption wavelength. This is consistent with the presence of multiple emitting species, as revealed by fluorescence lifetime measurements (*vide infra*). In Tris, there is a clear discrepancy between the long-wavelength edges of the excitation and absorption spectra, signifying the presence of non-emitting species that absorb at slightly longer wavelengths. In both solvents, the emission spectrum was independent of excitation wavelength.

As shown in Table 1, the excitation spectrum in Tris is blue-shifted relative to that in dioxane, whereas the emission spectrum is red-shifted, resulting in an increase in Stokes shift from 5400 cm^−1^ in dioxane to 8500 cm^−1^ in Tris. The high quantum yield of 0.78 in dioxane is reduced dramatically to 0.01 in Tris.

**Table 1:** Wavelengths of maximum spectral intensities (*λ*_max_), Stokes shifts (between excitation and emission maxima), and fluorescence quantum yields (*ϕ*) for MeO^th^aU as the free nucleoside and in oligonucleotides, AXA and AXT, single- and double-strands

Sample	*λ*_max_/nm			Stokes shift/cm^−1^	*ϕ* a
	Absorption	Excitation	Emission		
Nucleoside (dioxane)	384	376	472	5410	0.78
Nucleoside (Tris)	368	363	526	8540	0.01
AXA (SS)	386	375	495	6465	0.05
AXA (DS)	394	373	507	7085	0.03
AXT (SS)	388	375	491	6300	0.07
AXT (DS)	394	375	517	7325	0.03

a Quantum yields were measured at the absorption *λ*_max_ for the free nucleosides and for excitation at 394 nm for the oligonucleotides. Estimated uncertainty in *ϕ* values is 10%, except for the nucleoside in Tris where it is 20%.

The fluorescence decays of MeO^th^aU in each solvent were measured at three emission wavelengths and fitted globally with common lifetimes. Decay parameters are summarised in Table 2 and given in full in Tables S2 and S3 (ESI†).

**Table 2:** Fluorescence decay parameters for MeO^th^aU nucleoside in dioxane (excitation at 360 nm) and Tris buffer (excitation at 390 nm)

Solvent	*τ*_1_/ns^a^	*τ*_2_/ns	*τ*_3_/ns	*A*_1_ b	*A*_2_	*A*_3_	〈*τ*〉/ns
Dioxane	—	0.30	4.2	—	0.32	0.68	3.0
Tris	0.11	0.18	0.47	0.18	0.80	0.02	0.17

a Fluorescence lifetimes, *τ*_*i*_, were obtained by global fitting of decays recorded at three emission wavelengths.

b Fractional amplitudes, *A*_*i*_, and number-average lifetime, 〈*τ*〉, are quoted at an emission wavelength of 490 nm for dioxane and 525 nm for Tris buffer.

In dioxane, a biexponential decay with lifetimes of 0.30 ns and 4.2 ns was observed. The emitting population is dominated by the 4.2 ns component, with a fractional amplitude (*A*-factor) of 0.68 at 490 nm. The steady state emission intensity is due almost exclusively to this species (97% contribution) over the range 490 to 510 nm. In Tris, the decay becomes tri-exponential and the lifetimes are much shorter, 0.11 ns, 0.18 ns and 0.47 ns. The 0.18 ns component dominates the emitting population (A-factor of 0.8 at 525 nm) and accounts for the majority of the steady-state emission in the range 505 to 545 nm, increasing in contribution from 76% to 90% with increasing wavelength. The decrease in average lifetime from 3.0 ns in dioxane to 0.17 ns in Tris qualitatively reflects the decrease in quantum yield; however, the ratio of average lifetimes is substantially greater than the ratio of quantum yields, indicating the presence of non-emitting states. On the basis of these values, assuming the absence of non-emissive states in dioxane, we estimate that about 60% of the excited state population in Tris is non-emissive (see ESI† for details).

In its complex decay behaviour and the existence of non-emissive states in aqueous solution, MeO^th^aU resembles DMA^th^aU,^23^ although its non-emitting population is significantly lower than the 96% estimated for the latter. In both cases, two emitting species are observed in dioxane, whereas three emissive species and at least one non-emissive species are present in Tris. As discussed previously,^23^ this photophysical complexity can be related to the population of two distinct rotamers (with relative rotation of the aza-uridine and thiophene rings by 180°) each of which has three potential tautomers (Fig. 1). It seems likely that the two decay components seen in dioxane are due to the two rotamers, while the additional emitting and non-emitting species seen in aqueous conditions are due to the presence of tautomers that are not populated in the non-polar solvent.

#### MeO^th^aU-containing oligonucleotides

**Fig. 3:**
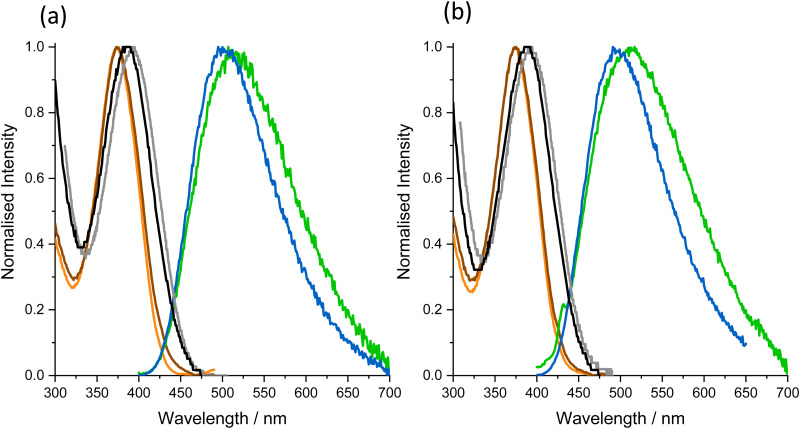
Normalised spectra for the oligonucleotides as single and double strands. (a) Oligonucleotide AXA. (b) Oligonucleotide AXT. Absorption spectra of single strand (black) and double strand (grey); excitation spectra, at emission wavelength 500 nm, of single strand (orange) and double strand (brown); emission spectra at excitation wavelength 375 nm, of single strand (blue) and double strand (green).

The absorption, excitation and emission spectra of the oligonucleotides, AXA and AXT, are shown in Fig. 3, for both single strand (SS) and double strand (DS), with *λ*_max_ values given in Table 1. The spectra of the two oligonucleotides are essentially identical, unaffected by the sequence context. For each oligo, both SS and DS, there is a sizeable discrepancy between the red edge of the absorption spectrum and that of the corresponding excitation spectrum, implying a significant non-emitting population absorbing at longer wavelengths than the emitting species. The excitation spectra of the SS and DS are essentially identical, while the absorption maximum of the DS shows a slight red shift relative to that of the SS. The emission spectrum of the DS is red-shifted and broadened compared with that of the SS.

The excitation spectra of the oligos are essentially identical to that of the nucleoside in dioxane, suggesting that the emission originates from similar ground-state species. The discrepancy between the absorption and excitation spectra for the oligos is much greater than for the free nucleoside, clearly indicating the presence of spectroscopically distinct, non-emitting species. The emission spectra of both SS and DS are red-shifted relative to the nucleoside in dioxane, but blue-shifted relative to Tris. The Stokes shift values of the oligos are intermediate between those of the nucleoside in the two solvents. It has been shown previously^21^ that the Stokes shift of the nucleoside shows a linear correlation with the empirical solvent polarity parameter, *E*_T_(30). Based on this correlation, we estimate that the local (average) environmental polarity experienced by the emitting species in the oligos corresponds to *E*_T_(30) values of 48 and 53 kcal mol^−1^, for SS and DS, respectively. For comparison, the *E*_T_(30) values of dioxane and water are 36 and 63 kcal mol^−1^, respectively.^25^ In the duplex, the methoxyphenyl arm of MeO^th^aU will lie in the major groove (like the methyl group of thymine). Previously, a responsive FBA related to MeO^th^aU has been used to probe the polarity of the major groove.^26^ This analogue of thymine, in which the methyl group is replaced by furan, returned an *E*_T_(30) value of 46 kcal mol^−1^. In this case, the furan group would be located close to the inner surface of the groove, whereas the aryl sidechain of MeO^th^aU will sample an environment further from the groove wall. As noted by Sinkeldam *et al.*,^26^ other studies, using polarity-sensitive fluorescent dyes attached by relatively long, flexible linkers to a natural base, report higher major groove polarity, with *E*_T_(30) values of around 58 kcal mol^−1^.^27–29^ For the SS oligos, our *E*_T_(30) value agrees with that of 48 kcal mol^−1^ reported by Sinkeldam *et al.*^26^ for the 13-mer used in their study. It is evident that, in both cases, the secondary structure of the single strand protects the FBA from the external aqueous environment. In the case of MeO^th^aU this results in a local environment that is less polar than that seen in the double strands.

The similarity of the excitation spectra of the free MeO^th^aU in dioxane, and in the SS and DS oligos can be rationalised in terms of the *E*_T_(30) values. As observed previously, the excitation spectrum of the nucleoside is unaffected by polarity in the range *E*_T_(30) = 45 to 54 kcal mol^−1^ (in dioxane–methanol mixtures).^21^ The influence of environmental polarity is manifested only in the red shift in the emission spectrum, which gives rise to the increasing Stokes shift with increasing *E*_T_(30), DS > SS > nucleoside in dioxane.

The quantum yield of MeO^th^aU in the oligos (Table 1) is greatly reduced (by more than an order of magnitude) from that in dioxane, although it is substantially higher (up to 7×) than in Tris. This is due in part to the decrease in quantum yield of the emitting species with increasing solvent polarity, but also reflects the appearance of non-emitting states, which were not observed in dioxane. Further insight into the nature of the emitting states and the size of the non-emitting population in the oligos is provided by the time-resolved fluorescence measurements discussed below. Although the quantum yield of 0.03 in DS oligos is lower than we might have hoped, it still exceeds that of many FBAs in duplex DNA.^4^

The fluorescence lifetimes of SS and DS oligos, obtained by global analysis across three emission wavelengths, are shown in Table 3, and the corresponding fractional amplitudes, as a function of emission wavelength, in Table 4.

**Table 3:** Fluorescence lifetimes, *τ*_*i*_, for single-stranded (SS) and double-stranded (DS) oligos, excited at 390 nm. (The corresponding fractional amplitudes are given in Table 4)

Oligo	*τ*_1_/ns^a^	*τ*_2_/ns	*τ*_3_/ns	*τ*_4_/ns
AXA SS	—	0.23	1.2	2.6
AXT SS	—	0.22	1.3	2.6
AXA DS	0.11	0.48	1.7	3.6
AXT DS	0.10	0.26	1.8	3.4

a Fluorescence lifetimes were obtained by global fitting of decays recorded at three emission wavelengths (470, 490, 510 nm).

**Table 4:** Fractional amplitudes (*A*-factors), *A*_*i*_, fractional contributions to the steady-state intensity, *S*_*i*_, and average lifetimes, 〈*τ*〉, for single-stranded (SS) and double-stranded (DS) oligos, as a function of emission wavelength, for excitation at 390 nm. The values of *S*_*i*_ were calculated using equation 3 in Section 1.2 of the ESI. (The corresponding lifetimes are given in Table 3)

Oligo	*λ*_em_/nm	*A*_1_	*A*_2_	*A*_3_	*A*_4_	*S*_1_	*S*_2_	*S*_3_	*S*_4_	〈*τ*〉/ns
AXA SS	470	—	0.28	0.55	0.17	—	0.05	0.58	0.37	1.18
	490	—	0.23	0.62	0.15	—	0.04	0.63	0.33	1.20
	510	—	0.26	0.44	0.30	—	0.05	0.65	0.30	1.15
AXT SS	470	—	0.29	0.45	0.26	—	0.04	0.41	0.55	1.42
	490	—	0.35	0.43	0.22	—	0.05	0.44	0.51	1.35
	510	—	0.25	0.62	0.13	—	0.06	0.46	0.48	1.24
AXA DS	470	0.29	0.11	0.52	0.08	0.03	0.04	0.68	0.25	1.25
	490	0.41	0.15	0.39	0.05	0.05	0.07	0.68	0.20	0.94
	510	0.56	0.16	0.25	0.03	0.09	0.12	0.63	0.16	0.66
AXT DS	470	0.29	0.12	0.49	0.10	0.02	0.03	0.69	0.26	1.26
	490	0.36	0.28	0.31	0.05	0.04	0.09	0.66	0.21	0.84
	510	0.39	0.41	0.17	0.03	0.07	0.19	0.56	0.18	0.55

For both SS and DS, the decay parameters of the two sequences, AXA and AXT, are very similar. The SS oligos show three decay components with lifetimes that are similar in magnitude to the two components seen for the nucleoside in dioxane, consistent with a relatively non-polar environment. The increase in the number of decay components in the oligos can be attributed to the heterogeneity of the local environment of MeO^th^aU, that results from the conformational mobility of the strands. The DS oligos show three decay times that are very similar to those of the SS, but with an additional shorter component of 0.1 ns, comparable to the shortest lifetime of the nucleoside in Tris. This suggests greater environmental heterogeneity in the duplex, with some conformational fluctuations resulting in aqueous exposure of MeO^th^aU.

The distribution of the emitting population among the lifetime components differs significantly between SS and DS oligos, as shown by the *A*-factors in Table 4. In the SS, *τ*_3_ (1.2 ns) accounts for about 50% of the emitting population across the emission wavelength range, with the other 50% distributed approximately evenly between *τ*_2_ (0.2 ns) and *τ*_4_ (2.6 ns). This results in an average lifetime of around 1.2 ns, lower than that of the nucleoside in dioxane (3.0 ns), but much higher than that in Tris (0.17 ns). In the DS, *τ*_3_ constitutes 50% of the emitting population at 470 nm, but at 510 nm, this has fallen to 25% in AXA and 17% in AXT, with the combined populations of the two sub-nanosecond species, *τ*_1_ and *τ*_2_, constituting 72% in AXA and 80% in AXT. In the DS, *τ*_4_ is a minor component, constituting 10% of the emitting population at 470 nm and only 3% at 510 nm. In the DS, there is a higher population of shorter-lifetime, longer-wavelength emitting species, reflecting access of MeO^th^aU to higher-polarity microenvironments. This leads to a marked wavelength-dependence of the average lifetime for the DS, which decreases from 1.25 ns (as high as the SS) at 410 nm to 0.66 ns for AXA (0.55 ns for AXT) at 510 nm.

We also examined the contribution of the different emitting species to the steady-state emission spectra (*S*_*i*_ values in Table 4). For the SS, the emission intensity is due almost entirely (>94%) to the two longer-lifetime species, *τ*_3_ and *τ*_4_, both of which contribute substantially. For the DS, the steady-state intensity is dominated by *τ*_3_ across the measured wavelength range, but the combined contribution of the short-lifetime species, *τ*_1_ and *τ*_2_ becomes increasingly significant with increasing wavelength (>20% at 510 nm). The emission from the short-lifetime species can be correlated with the red shift in the DS emission spectrum relative to that of the SS (Fig. 3), and the higher average polarity reported by the Stokes shift.

For both SS and DS, the evidence of non-emissive states seen in the discrepancy between steady-state absorption and excitation spectra (Fig. 3) is confirmed by the lifetime measurements. The average lifetimes are decreased by about a factor of 2 compared with the nucleoside in dioxane, but the quantum yields are decreased by a factor of 10 to 20. On this basis, using average lifetime values measured at 510 nm, we estimate the fractional non-emitting populations of the SS oligos to be AXA ∼70% and AXT ∼60% and, for the DS, ∼50% in each case (see ESI† for details of calculation). The similar magnitudes of these populations across the oligos and the nucleoside in Tris suggests that, in all cases, the same non-emitting species are present and are intrinsic to the nucleoside, rather than being the consequence of inter-base quenching in the oligos. We attribute these non-emissive states to enol tautomers of the aza-uridine core (Fig. 1). In the oligos, although MeO^th^aU is shielded from the bulk aqueous solvent, the tautomers can be stabilised by specific interactions with water molecules. Even in base-stacked conformations of the duplex, the carbonyl oxygens (equivalent to O2 and O4 of thymidine), will be exposed to water molecules in the minor and major grooves, respectively.

The fluorescence decay parameters of MeO^th^aU in the SS and DS oligos can be rationalised in terms of the influence of the microenvironmental polarity. We see no evidence of inter-base quenching, unlike other responsive FBAs such as 2AP and ^th^G. However, more extensive investigation of other sequence contexts is required to confirm this. Several other polarity-sensitive FBAs have been reported previously;^5^ typically, these are extended FBAs comprising a known environment-sensitive push–pull fluorophore tethered to a nucleoside core *via* a flexible non-conjugating linker. To our knowledge, their response to incorporation in oligonucleotides has been characterised only in terms of their emission wavelength and quantum yield, not their fluorescence decay properties.

When considering the feasibility of pushing the detection limit to the single-molecule level, it is informative to consider the quantum yields of the individual emitting species, rather than the average value (from steady-state measurements) that includes non-emissive states. The quantum yield of an emitting species can be predicted from its direct relationship to the fluorescence lifetime of that species. The species-specific quantum yields were calculated with reference to the measured quantum yield of the nucleoside in dioxane (equation S7 in ESI†); since there are no non-emitting species in dioxane, the average lifetime of the nucleoside is directly proportional to the average quantum yield (equation S2 in ESI†). Details of the calculation are given in Section S3 of the ESI.† The species-specific quantum yields are given in Table 5 for AXA single and double strands. The values for the AXT oligos are essentially identical. In both SS and DS, the quantum yields of the two longest lifetime components, *ϕ*_3_ and *ϕ*_4_, are relatively high, 0.2 and 0.3, respectively. Together, these two brightly emitting species constitute about 70% of the emitting population (20% of the total population) in the SS and about 40% (20%) in the DS. These are the species that we might expect to be detected in the FCS measurements.

**Table 5:** Quantum yields of the individual emitting species for AXA single and double strands, estimated from lifetime values as described in Section 2.3 of the ESI

Oligo	*ϕ*_1_	*ϕ*_2_	*ϕ*_3_	*ϕ*_4_
AXA SS	—	0.03	0.2	0.3
AXA DS	0.01	0.05	0.2	0.3

### 2P photophysics

#### Free MeO^th^aU nucleoside

The two-photon brightness values of the free nucleoside in dioxane and tris were measured at an excitation wavelength of 780 nm. In each case, the log–log plot of emission intensity *versus* laser power shows a gradient of 2, confirming a two-photon absorption process (Fig. S5 and S6, ESI†). In dioxane, the emission spectra under 1P and 2P-excitation are identical (Fig. S7, ESI†), suggesting that the same emitting population is being observed in both cases. This supports the use of the average quantum yield, measured under 1P-excitation, to infer the value of 13 GM for the 2P cross-section (Table 6). However, in Tris, there is a clear discrepancy between 1P- and 2P-excited emission spectra (Fig. S8, ESI†), implying different emitting populations in each case; therefore, we do not quote a value for the 2P cross-section in Tris.

**Table 6:** Two-photon brightness (*σ*_2_*ϕ*) and two-photon cross-section (*σ*_2_), at an excitation wavelength of 780 nm, for the nucleoside in Tris and dioxane, and for the oligos as single and double strands

Sample	*σ*_2_ *ϕ*/GM^a^	*σ*_2_/GM^b^
Nucleoside (dioxane)	10	13
Nucleoside (tris)	0.05	—
AXA (SS)	0.66	13
AXA (DS)	0.31	8.0
AXT (SS)	0.82	12
AXT (DS)	0.25	6.2

a Estimated uncertainty is 10%.

b Cross-section values were calculated using quantum yields measured at excitation wavelengths of 384 nm for the free nucleoside, and 394 nm for the oligonucleotides. Estimated uncertainty is 15%.

As shown in Table 6, the two-photon brightness of the free nucleoside in Tris (0.05 GM) is much lower than that in dioxane (10 GM), as might be expected given the much lower quantum yield in Tris. We also note that the 2P excitation wavelength of 780 nm lies closer to the one-photon absorption maximum in dioxane than that in Tris, so the 2P cross-section at this wavelength is likely to be higher in the former case.

#### MeO^th^aU-containing oligonucleotides

The two-photon brightness of each oligo was measured as a function of excitation wavelength over the range 760 to 800 nm, as shown in Table S4 (ESI†). The maximum value was obtained for 780 nm excitation in each case; these values are given in Table 6. For all measurements, log–log plots of emission intensity *versus* laser power confirmed 2-photon absorption (Fig. S9 and S10, ESI†). As shown in Fig. S11 (ESI†), for all the oligos, the emission spectra under 1P-and 2P-excitation are in close agreement, supporting the use of 1P-excited quantum yields to extract cross-section values.

The 2P cross-section of MeO^th^aU in the SS oligos (13 GM, 12 GM) is, within experimental error, unchanged compared with the free nucleoside in dioxane, but in the DS oligos it is reduced to 8.0 GM for AXA (about 60% of the SS value) and about 6.2 GM for AXT (about 50% of the SS value). The 2P cross section depends on both the transition dipole moment and the change in permanent dipole moment (Δ*μ*) on excitation (in a two-level model for a dipolar chromophore).^30^ Incorporation of MeO^th^aU in the SS oligos results in a small decrease in its one-photon molar absorption coefficient (indicative of the transition dipole moment) by about 10%. In the DS, the molar absorption coefficient is further reduced to about 70% of the value in the SS. The trend in 2P cross-sections correlates well with the trend in the absorption coefficients, suggesting that there is no significant effect of the molecular environment on Δμ. This contrasts with our observations for pA in oligos, where we found a much greater decrease in 2P cross-section than 1P absorption coefficient, an effect that could be attributed to a decrease in Δμ caused by the local electrostatic field.

**Fig. 4:**
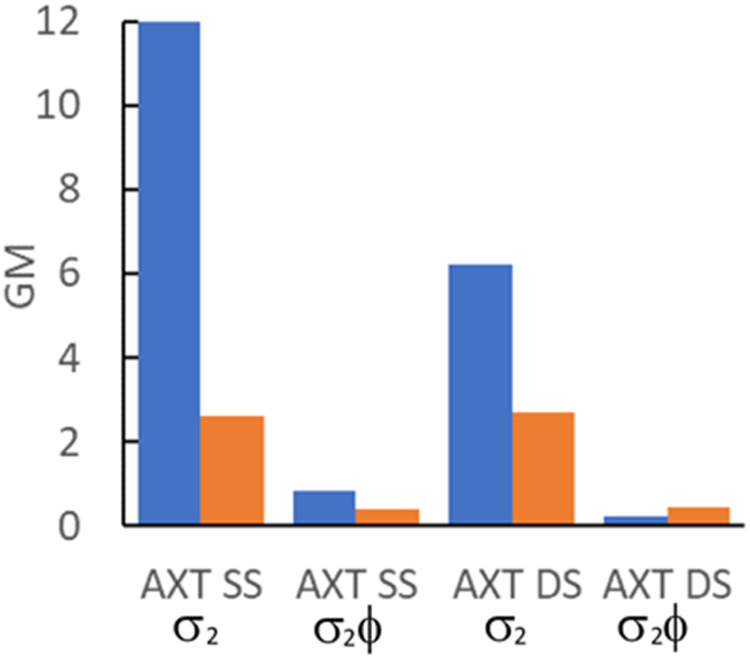
Comparison of 2P cross-section (*σ*_2_) and 2P brightness (*σ*_2_*ϕ*) values for AXT oligos where X = MeO^th^aU (blue) or pA (orange).

The 2P cross-sections of MeO^th^aU in the oligonucleotides are significantly higher than those we reported previously for pA, which showed values in the range 2.4 to 3.0 GM across several sequences of SS and DS oligos,^20^ with little difference between SS and DS. A comparison of cross-sections and brightness values between AXT and a pA-containing oligo, with an identical sequence (with X = pA), is shown in Fig. 4.

In the context of detection sensitivity, the 2P brightness is the crucial parameter. Unfortunately, the promising cross sections of MeO^th^aU in the oligos are somewhat negated by the relatively low quantum yields. In SS AXT the brightness of MeO^th^aU, 0.82 GM, compares favourably with that of pA, 0.39 GM, but in DS AXT the brightness of MeO^th^aU, 0.25 GM, is lower than that of pA, 0.46 GM. While we are comparing here 2P brightness values for MeO^th^aU and pA in the same oligo sequence context, it should be mentioned that the quantum yield of pA in SS oligos, and hence its brightness, depends strongly on its nearest-neighbour bases.^20^ We measured a maximum value of 1.3 GM for a SS with pA flanked by G and A and a minimum value of 0.06 GM for pA flanked by T on each side. In DS oligos, the quantum yield of pA depends only weakly on context, and the 2P brightness for AXT was the highest measured.

To our knowledge, there have been no other previous measurements of 2P cross-sections of FBAs in DNA oligos. However, Nilsson *et al.*^24^ recently reported a cross-section of 6.9 GM (at 700 nm) for the quadracyclic adenine analogue 2CNqA in a 16mer antisense oligonucleotide (ASO). An ASO is a single-stranded deoxyribonucleotide, with a modified phosphorothioate backbone, which is complementary to an RNA target. Using the quantum yield of 0.22 reported previously for 2CNqA in this ASO,^31^ gives a 2P brightness of 1.52 GM, about twice that of MeO^th^aU in SS AXT and comparable to the maximum value of 1.3 GM for pA in a SS oligo.

The 2P brightness values discussed here are, of course, values averaged over all absorbing species. As already shown (Table 5), there are sub-populations of MeO^th^aU emitting species in the oligos that have quantum yields that are an order of magnitude greater than the average value. Therefore, we would expect that there will be MeO^th^aU-containing duplexes with sufficiently high 2P brightness to be detectable at the level of a few molecules in FCS experiments.

### Pulse-shaped 2P microscopy

**Fig. 5:**
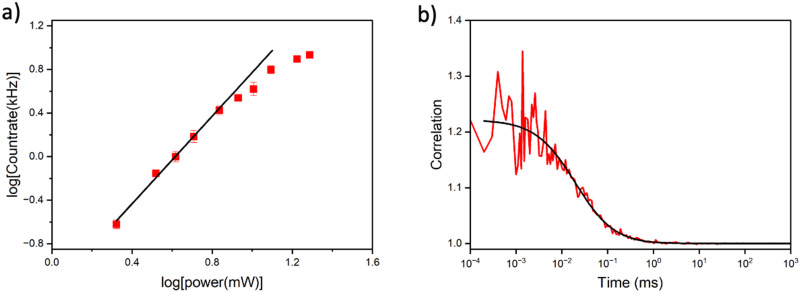
(a) Power dependency measurements of 100 nM MeO^th^aU nucleoside in Tris. The gradient of the linear region is 2.0, with the onset of saturation around 9 mW. (b) FCS measurement of 250 nM MeO^th^aU nucleoside in Tris. The excitation power was 9.6 mW. The red line is the measured correlation curve; the black line is the fit to diffusion through a 3D Gaussian volume with 1.5 molecules on average in the focus. The buffer background intensity was 0.32 kHz.

Multiphoton microscopy of MeO^th^aU as the nucleoside and in the AXA and AXT oligos (SS and DS) was performed in Tris buffer. The excitation source was a broadband Ti:sapphire laser, as used previously for FBAs.^13,20,23^ By correcting for dispersion in the optical setup, the sample is irradiated with laser pulses of ∼10 fs, allowing efficient multiphoton excitation.^32^ As discussed, MeO^th^aU nucleoside has an absorption maximum at 368 nm in Tris (Table 1). Plotting the absorption of the MeO^th^aU nucleoside *versus* twice the wavelength shows good overlap with the broadband laser spectrum suggesting it would be a good candidate for 2P excitation (Fig. S12, ESI†). Measuring fluorescence intensity as a function of laser power confirmed that 2P excitation occurs under this experimental regime. At lower laser powers, the log–log plot (Fig. 5a) shows a linear relationship with a gradient of 2.0. Unlike DMA^th^aU,^23^ no three-photon absorption was observed at higher laser powers, with evidence of saturation in MeO^th^aU emission from around 9 mW.

FCS measurements of the MeO^th^aU nucleoside were performed with 9.6 mW excitation power using the full spectral bandwidth of the laser (Fig. 5b). The correlation curve was fitted to a model that assumes that intensity fluctuations are due to 3D Gaussian diffusion (equation S4 in the ESI†). The countrate per molecule (CPM) of the nucleoside was measured to be 3.7 ± 0.5 kHz, which is approximately half that of DMA^th^aU.^23^ The diffusion time was 25 ± 5 μs, the same as the value reported for DMA^th^aU, which was measured under identical conditions using the same microscope.^23^ Interestingly, there appeared to be far fewer molecules in the detection volume than expected (0.6 for a 100 nM sample), pointing to the presence of non-emissive states. Therefore, a controlled dilution was performed, with the number of molecules in the detection volume scaling linearly with concentration (Fig. S13, ESI†). By comparing with rhodamine 110, and making the assumption that every rhodamine 110 molecule is detected, this suggests that ∼4% of the nucleoside molecules exist in a bright state. The remaining 96% are assigned to non-emissive species. This is much greater than the non-emitting fractional population of 62% estimated from the 1P measurements (*vide supra*). This suggests that FCS is only detecting the brightest emissive species observed by time-resolved ensemble fluorescence (*i.e.* the species with a lifetime of 0.47 ns in Table 2, which only constituted 2% of the observed molecules following 1P excitation). As already noted (*vide supra*), the emission spectrum of the nucleoside recorded under 2P-excitation differs from that recorded under 1P-excitation (Fig. S8, ESI†), so we do not expect perfect correspondence between the 1P- and 2P-excited populations.

Single-molecule detection was attempted using multichannel scaling (MCS) to detect photon bursts from single molecules diffusing through the laser focus (Fig. S14, ESI†). The highest photon event for the nucleoside was only 11 photons compared to the 10 photons for the buffer, indicating that reliable single-molecule bursts are not discernible above the background level. This is consistent with our previous findings for DMA^th^aU, with a brightness of 7 kHz, where single-molecule bursts were only just detectable.^23^

**Fig. 6:**
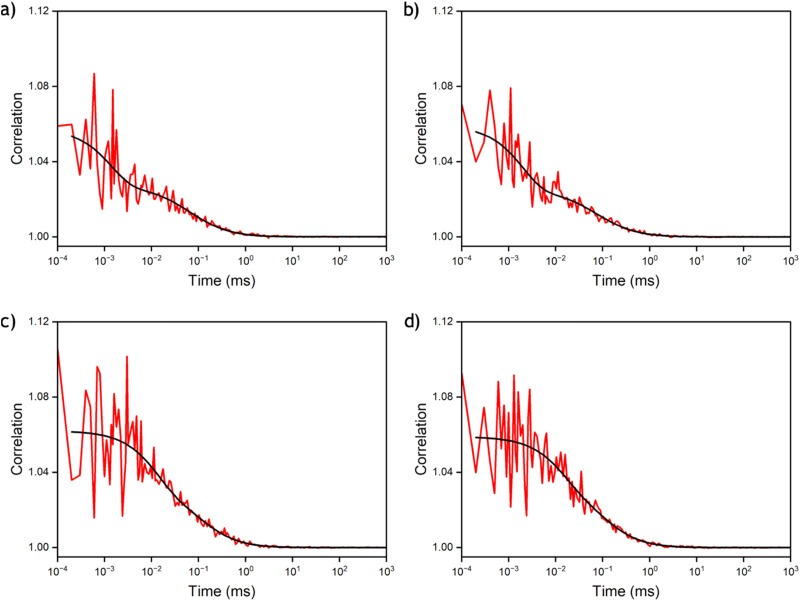
FCS measurements of MeO^th^aU containing oligonucleotides (100 nM) at 9.6 mW excitation power. (a) AXA SS. (b) AXT SS. (c) AXA DS. (d) AXT DS. The red lines are the measured correlation curves; the black lines are the fits to 3D diffusion through a Gaussian volume, along with an additional dark state.

FCS was subsequently used to measure MeO^th^aU in the four oligonucleotide sequences: AXA SS, AXA DS, AXT SS and AXT DS (Fig. 6). From the FCS measurements, between 7 and 12 molecules can be detected in the focus (Table 7), which is in good agreement with the number expected from controlled dilution of the stock solution. Good fits were achieved using a model (equation S5 in the ESI†) that includes an additional component for a dark state with a lifetime shorter than the diffusion time (Table 7). The results were similar for both oligos. As an example, AXA SS was measured to have a CPM of 0.9 ± 0.06 kHz with a diffusion time of 70 ± 5 μs, while 46 ± 6% of the molecules were in a dark state with a lifetime of 3 ± 3 μs. Moving from the single strand to the duplex there was little difference in the CPM of AXA DS at 0.8 ± 0.09 kHz, though there was a small increase in the diffusion time to 83 ± 13 μs due to the increased molecular weight of the sample. The dark state fraction in the AXA DS decreased slightly to 41 ± 9%, with an increase in the lifetime of this state to 8 ± 5 μs. In general, the dark state populations observed for the AXA and AXT samples (Table 7), agree well with the estimates from 1P excitation (*vide supra*), meaning that emissive MeO^th^aU converts to the non-emissive species during its transit through the laser focus. The apparent discrepancy between relative 2P brightness for nucleoside *versus* oligos measured by FCS and ensemble methods can be accounted for by the fact that the ensemble approach is an average over all the molecules (emissive and non-emissive), while FCS is only detecting the bright emissive species. The observed CPM values make MeO^th^aU the brightest FBA measured *via* multiphoton excitation after incorporation into duplex DNA.

**Table 7:** Parameters extracted from FCS measurements of the MeO^th^aU oligonucleotides. The data were fitted to eqn (S5) in the ESI. The values and errors presented are the average and standard deviation, respectively from three measurements

Oligo	*N*	CPM/kHz	*τ*_d_/μs	Dark state fraction	Dark state lifetime/μs
AXA SS	11.8 ± 0.7	0.9 ± 0.06	70 ± 5	0.46 ± 0.06	3 ± 3
AXA DS	7.3 ± 0.9	0.8 ± 0.09	83 ± 13	0.41 ± 0.09	8 ± 5
AXT SS	12.0 ± 1	0.8 ± 0.07	68 ± 11	0.55 ± 0.04	2 ± 0.4
AXT DS	7.6 ± 0.3	0.8 ± 0.01	82 ± 2	0.35 ± 0.01	10 ± 5

## Conclusions

The photophysical properties of MeO^th^aU in oligonucleotides are influenced primarily by the polarity of the local environment. Unlike many responsive FBAs, its fluorescence does not appear to be quenched by inter-base interactions. The sensitivity of its emission wavelength, quantum yield and fluorescence decay parameters to variations in the polarity of the microenvironment within oligonucleotides make MeO^th^aU a potentially valuable probe of conformational changes or external interactions that modulate the ingress of water molecules into the DNA structure.

In aqueous solution, the free nucleoside exhibits non-emitting states, constituting 60% of the excited-state population, which we attribute to enol tautomers. In oligonucleotides, this non-emitting population is maintained, but there is a substantial increase in the quantum yield of the emitting species in the less polar environment. The average lifetime of MeO^th^aU increases by a factor of 7 on going from Tris (0.17 ns) to DS DNA (1.25 ns), a range of lifetimes easily measurable with a standard time-correlated single-photon counting system equipped with a 400 nm pulsed diode laser. Moreover, the DS can be distinguished from the SS by the appearance of an additional short-lifetime component (0.1 ns) and a marked dependence of the average lifetime on emission wavelength. MeO^th^aU would thus be particularly suitable for fluorescence lifetime-based sensing and imaging (FLIM) applications.

The 2P cross-sections of MeO^th^aU in oligonucleotides exceed those reported previously for pA, by a factor of 4 for SS and factor of 2 for DS, but the 2P brightness, averaged over all absorbing species (emitting and non-emitting), compares less favourably with pA. However, there are sub-populations of MeO^th^aU emitting species in the oligos that have quantum yields of around 0.3, exceeding the average value by as much as an order of magnitude. The existence of these brightly fluorescent species enabled the detection of as few as 7 molecules of MeO^th^aU-containing duplex oligonucleotides, using pulse-shaped 2-photon excitation. This illustrates the importance of considering the heterogeneity of the excited-state population when assessing the potential of an FBA for single-molecule detection. It is not uncommon for FBAs to exhibit non-emitting states, and the average quantum yield may not be a reliable indicator of the brightness of individual emitting species.

The work reported here provides further evidence that 2-photon excitation, enhanced by pulse-shaping, is an effective strategy for the ultrasensitive detection of FBAs. The detection of less than 10 molecules of a responsive FBA within duplex DNA is an important step towards the ultimate goal of elucidating DNA dynamics and the mechanisms of DNA–enzyme processes at the single-molecule level, with single-base resolution.

## Data availability

The data supporting this article have been included as part of the ESI.†

## Conflicts of interest

There are no conflicts to declare.

## Supplementary Material

CP-026-D4CP03391D-s001
