# Supplementary material for: Ultrasensitive detection of a responsive fluorescent thymidine analogue in DNA via pulse-shaped two-photon excitation
Source: Phys Chem Chem Phys. 2024 Oct 8;26(42):26823–33. doi: 10.1039/d4cp03391d (PMC11476554; doi:10.1039/d4cp03391d)
Supplement: CP-026-D4CP03391D-s001 [file CP-026-D4CP03391D-s001.pdf]

Supplementary Information (SI) for Physical Chemistry Chemical Physics.
This journal is © the Owner Societies 2024

Ultrasensitive detection of a responsive fluorescent thymidine analogue in DNA *via* pulse-shaped two-photon excitation

Alexandra E. Bailie, Henry G. Sansom, Rachel S.Fisher, Ryo Watabe, Yitzhak Tor, Anita C. Jones* and Steven W. Magennis*

Supplementary Information

S1 Experimental

S1.1 Materials

Synthesis of oligonucleotides

The synthesis and characterization of the MeOthaU nucleoside has been reported previously.[1]

The phosphoramidite was synthesized using the following procedure. The MeOthaU nucleoside (71.3 mg, 0.171 mmol, 1.0 eq) was co-evaporated with pyridine twice and dried in vacuo for 1 hr. 4,4'-Dimethoxytrityl chloride (115.7 mg, 0.342 mmol, 2.0 eq) was added to the nucleoside. The mixture was dried in vacuo for 3 hrs and then dissolved in pyridine (0.85 mL). The solution was stirred at room temperature for 17 hrs. The reaction was quenched with MeOH and evaporated. The residue was purified by column chromatography with 0 to 10% gradient of MeOH in DCM with 1% Et_3N. The product obtained was dissolved in DCM and washed three times with *aq.* $NaHCO_3$, dried over Na_2SO_4 and then evaporated to afford a yellow solid (98.9 mg, 0.137 mmol, 80% yield). ^1H NMR (500 MHz, $CDCl_3$) δ 10.09 (s, 1H), 7.95 (d, *J* = 5.0 Hz, 1H), 7.56 (d, *J* = 15.0 Hz, 2H), 7.40–7.33 (m, 2H), 7.08–7.25 (m, 8H), 6.93 (d, *J* = 15.0 Hz, 2H), 6.70–6.64 (m, 4H), 6.63–6.58 (m, 1H), 4.88 – 4.75 (m, 1H), 4.13 – 4.00 (m, 1H), 3.86 (s, 3H), 3.68 (s, 6H), 3.50 – 3.38 (m, 1H), 3.31 – 3.19 (m, 1H), 2.75 – 2.62 (m, 1H), 2.43 – 2.30 (m, 2H); MS (ESI-TOF) m/z: $[M + Na]^+$ calcd. for $C_{40}H_{37}N_3O_8SNa^+$ 742.22 , found 742.22.

The DMTr-protected MeOthaU nucleoside (45.4 mg, 0.0631 mmol, 1.0 eq) was co-evaporated three times with pyridine, dried in vacuo overnight and then dissolved in DCM 0.32 mL. DIPEA (0.044 mL, 0.252 mmol, 4.0 eq) and 2-cyanoethyl *N*,*N*-diisopropylchlorophosphoramidite (24.8 mL, 0.105 mmol, 1.7 eq) were successively added to the solution and the mixture was stirred at room temperature for 2.5 hrs. The reaction mixture was diluted with EtOAc and evaporated. The residue was purified by column chromatography with 0 to 10% gradient MeOH in DCM with 1% Et_3N. The collected fractions were concentrated and then dissolved in DCM, followed by precipitation from hexane to isolate the product as yellow solid (26.4 mg, 0.0287 mmol, 45% yield). It was a mixture of two diastereomers. ^1H NMR (500 MHz, $CDCl_3$) δ 7.94 – 7.88 (m, 1H), 7.59 – 7.49 (m, 2H), 7.41 – 7.31 (m, 2H), 7.25 – 7.06 (m, 6H), 7.00 – 6.89 (m, 2H), 6.69 – 6.56 (m, 6H), 4.93 – 4.70 (m, 1H), 4.33 – 4.12 (m, 1H), 3.87 (s, 3H), 3.67 (s, 6H), 3.67 – 3.50 (m, 4H), 3.45 – 3.18 (m, 3H), 2.63 – 2.30 (m, 4H), 1.35 – 1.00 (m, 12H); ^{31}P (500 MHz, $CDCl_3$): δ 148.89, 148.33; MS (ESI-TOF) m/z: $[M + H]^+$ calcd. for $C_{49}H_{55}N_5O_9SP^+$ 920.35, found 920.37.

Bz-dA phosphoramidite, Bz-dC-phosphoramidite, iBu-dG-phosphoramidite and dT-phosphoramidite were purchased from Thermo Fisher Scientific and solid support (Glen UnySupport™ 500) was purchased from Glen Research. All the phosphoramidites, except MeOthaU phosphoramidite, were dissolved in anhydrous MeCN to a final concentration of 0.067 M.

The synthesis of oligonucleotides was performed on a 1.0 micromol scale by using an automated DNA synthesizer (BioAutomation, MerMade6) with 0.25 M 5-(benzylthio)-1H-tetrazole in MeCN (Glen Research) as an activator, 3% trichloroacetic acid in dichloromethane (Glen Research) as a deblocking reagent, 0.5 M CSO in MeCN (Glen Research) as an oxidizing reagent, and a combination of 5% phenoxyacetic anhydride in THF/pyridine (Glen Research) and 16% 1-methylimidazole in THF (Glen Research) as a capping reagent. The DNA chain assembly before incorporating MeOthaU was carried out with the automated synthesizer. The solid support was removed and dried in vacuo overnight and then transferred into an empty column for an incorporation of MeOthaU by manual coupling. The support was mixed with a solution of MeOthaU phosphoramidite in MeCN (0.14 M, 0.1 mL) and 5-(benzylthio)-1H-tetrazole in MeCN (0.25 M, 0.16 mL) for 30 min under nitrogen at ambient temperature followed by oxidation step with CSO solution (0.5 M, 1.5 mL) for 5 min. After the deblocking step was conducted, the support was washed with MeCN, dried in vacuo and then placed back on the automated synthesizer for the automated incorporation of the remaining residues. The oligonucleotides were cleaved and deprotected via treatment with 1 mL 30% NH_4OH at 50 degrees Celsius for 18 hrs. The solution was dried by SpeedVac. The obtained crude product was dissolved in 1 mL autoclaved water and then purified by polyacrylamide gel electrophoresis (PAGE).

The oligonucleotides AXA and AXT were characterised by LC-ESI-TOFMS (Agilent 6230 ESI-TOFMS coupled with Agilent 1260 HPLC), as shown in Figs. S1-4. The Jetstream electrospray ionization source was operated under negative ion mode and the chromatographic separation was performed on a Waters XBridge Oligo column (2.1 mm ID x50 mm length, 2.5 um particle size).

Complementary strands were purchased from ATD Bio Ltd.

Samples for ensemble 1P and 2P spectroscopy.

The free the MeOthaU nucleoside was dissolved in 1,4-dioxane (99.8 %, extra dry, Acros Organics) or Tris buffer (20 mM Tris with 150 mM NaCl, pH 7.8). The same Tris buffer was used to dissolve MeOthaU-containing oligonucleotides. Duplex oligonucleotides were annealed by combining each modified single strand with 10% excess of its complementary strand at RT followed by heating to 95 °C and leaving to cool overnight. Absorption spectroscopy was used to confirm annealing.

Samples for 2P microscopy

The nucleoside was initially dissolved in DMSO at a concentration of 20 mM and subsequently diluted in buffer for measurement. Tris buffer (20 mM Tris, 150 mM NaCl, pH 7.8) was prepared using ultrapure water trimza base (Merck, BioUltra), trimza HCl (Merck, BioUltra), and NaCl (Merck, BioUltra),. The pH was adjusted using 5 M HCl (Sigma-Aldrich). To remove fluorescent impurities the buffer was filtered with activated charcoal (Darco, Fluca) and through a 0.2 µm filter (Milex, Merck). MeOthaU-containing oligonucleotides were dissolved in this buffer and subsequently diluted for measurement.

S1.2 1P Spectroscopy

Absorption spectra were collected using a Shimadzu UV-2700 UV-visible spectrophotometer, running UV-2700 UVProbe 2.43 software, and fluorescence spectra were recorded using a Jobin Yvon Horiba FluoroMax-P spectrofluorimeter, running FluorEssenceTM software v.3.5. Samples for fluorescence measurements were of absorbance between 0.02 and 0.1 at the chosen excitation wavelength.

Fluorescence quantum yields were measured relative to a reference fluorophore, Coumarin 153 or Prodan in toluene. The integrated fluorescence intensity was measured as a function of absorbance, over a range between 0.02 and 0.1, for the sample and reference fluorophore. In the case of all double-stranded oligonucleotides, scarcity of material prevented the use of this method, so quantum yields were determined from measurement at a single concentration.

Fluorescence lifetimes were determined using time-correlated single-photon counting on an Edinburgh Instruments spectrometer equipped with TCC900 photon counting electronics. A mode-locked Ti:sapphire laser (Coherent Mira pumped by Coherent Verdi), producing pulses of duration ~ 150 fs at a repetition rate of 76 MHz, was used as the excitation source. The pulse repetition rate was reduced to 4.75 MHz using a pulse picker (Coherent 9200) and the light was frequency-doubled using a Coherent 5-050 harmonic generator. Fluorescence decay curves were recorded over 50 ns, in 4096 channels, and collected to a total of 10000 counts in the peak channel. Decays were fitted by by iterative re-convolution, assuming a multi-exponential function, given in eq 1.

$$I(t) = \sum_{i=1}^{n} A_i \exp\left(\frac{-t}{\tau_i}\right) \qquad (1)$$

where I is the fluorescence intensity as a function of time, t, (normalised to the intensity at t=0); τ_i is the fluorescence lifetime of the ith decay component and A_i is the fractional amplitude (A-factor) of that component. (The fractional amplitude depends on the radiative lifetime of that emitting species and the fraction of the emitting population that it constitutes). Decays were collected at three emission wavelengths and were analysed globally, with τ_i as the common parameters, using Edinburgh Instruments software FAST.

The average lifetime, $< \tau >$, of the emitting population is related to the average quantum yield, , $< \phi >$, determined from steady-state intensity measurements, as given by eq 2.

$$< \tau > = \frac{\sum_{i=1}^{n} A_i \tau_i}{\sum_{i=1}^{n} A_i} = \frac{<\phi>}{<k_r>} \qquad (2)$$

where $<k_r>$ is average radiative lifetime of the emitting population.

The fraction of the steady-state emission intensity due to each species i, S_i, is given by eq 3.

$$S_i = \frac{A_i \tau_i}{\sum_{i=1}^{n} A_i \tau_i} \quad (3)$$

S1.3 2P Spectroscopy

The fundamental output of a mode-locked Ti:sapphire laser (see above) was used as the excitation source. A variable reflective neutral density filter was used to attenuate the excitation beam, which then passed through a dichroic mirror (Semrock Brightline FF735-Di02) and was focused by a 10× objective into the sample solution, which was contained in a 1-cm path-length cuvette. Fluorescence emission was collected by the same objective, reflected from the dichroic mirror, passed through a shortpass filter (Semrock Brightline FF01-720/SP-25) and detected by a fibre-coupled spectrometer (Ocean Optics USB2000+). Spectra were measured in triplicate and corrected for the wavelength response of the spectrometer. The incident power was measured using a Coherent FieldMaster power meter. Two-photon cross-sections were measured relative to Coumarin 153 or Prodan in toluene. Integrated emission intensity was measured in triplicate at 10 different incident laser powers.

S1.4 2P microscopy

The two-photon microscope is a homebuilt pulse-shaper assisted setup with a ultra-broadband Ti:Sapphire laser as light source.[2] The broadband Ti:Sapphire laser had a repetition rate of 80 MHz and a spectrum centred on 800 nm with a FWHM of 135 nm (Vitara UBB, Coherent). The compressed pulses from the oscillator had a duration of 10 fs. To compensate for dispersion in the objective the pulses were compressed using the MIIPS method with a pulse shaper (Biophotonics, MIIPS-Box 640).[3] The beam was subsequently focused onto the sample by a 60x water-immersion objective (UPlanSApo, Olympus). The pulses at the sample had a duration of 7 fs. The nucleoside sample was placed on a cover slip (Menzel Gläser #1.5 thickness, Thermo Scientific). For the MeOthaU samples 1 mL of solution was placed in a 4-welled chamber slide (Nunc Lab-Tek II, Fisher). The solution temperature was controlled (22 ± 1°C) with an incubator (Live Cell Instrument, CU-501). Sample fluorescence was collected by the same objective and transmitted through a dichroic mirror (Chroma 675dcspxr) and a short-pass filter (Semrock FF01-650/SP), split by a polarising beamsplitter cube and detected by two avalanche photodiodes (MPD PDM 50c and MPD $PD-050-CTB). For the multichannel scalar measurements, two different avalanche photodiodes were used (Hamamatsu,C11202-050), with lower dark counts and increased sensitivity in the UV.

For fluorescence correlation spectroscopy (FCS), the signals from the APDs were cross-correlated using a hardware correlator (ALV-7002, ALV GmbH).

The correlation curve $G(t)$ was fitted with equation eq. 4 or eq. 5.

$$G(t) = \frac{\left(1 - \frac{I_B}{S}\right)^2}{\sqrt{8}N}\left(1 + \frac{t}{\tau_D}\right)^{-1}\left(1 + \frac{t}{k^2\tau_D}\right)^{-\frac{1}{2}} + 1 \quad (4)$$

Eq. 4 is for diffusion through a 3D Gaussian volume, and takes in to account the intensity of the

background, I_B, and the signal, S. N is the number of molecules in the focus, τ_D is the characteristic diffusion time and $k = z_0/\omega_0$, which are the height and waist of the excitation volume, respectively.

For samples with triplet or other dark states, an additional component was added to give eq. 5:

$$G(t) = \frac{\left(1 - \frac{I_B}{S}\right)^2}{\sqrt{8}N(1-D)}\left(1 + \frac{t}{\tau_D}\right)^{-1}\left(1 + \frac{t}{k^2\tau_D}\right)^{-\frac{1}{2}} * \left[1 - D + Dexp\left(-\frac{t}{\tau_{dark}}\right)\right] + 1 \qquad (5)$$

where D and τ_{dark} are the dark state fraction and lifetime, respectively.

The average number of molecules in the confocal volume was determined from these fits allowing calculation of the count-rate per molecule (CPM).

For MCS experiments the signals from the SPADs first passed through a router (HRT-82, Becker and Hickl) before being detected by a photon-counting card (SPC-130, Becker and Hickl).

S2 Calculation of population of non-emitting species

The presence of non-emitting species, i.e. species with fluorescence lifetimes too short to be detected at the experimental time resolution, can be inferred from a discrepancy between the relative steady-state quantum yield and the corresponding relative number-average lifetime.

The fractional population of dark states. A_0, is given by eq. 6

$$A_0 = 1 - \frac{<\phi>_{\mathrm{rel}}}{<\tau>_{\mathrm{rel}}<k_r>_{rel}} \qquad (6)$$

where $< \phi >_{\mathrm{rel}}$ is the relative, average quantum yield (measured, for example, in two different solvents), $< \tau >_{\mathrm{rel}}$ is the corresponding relative, number-average lifetime and $< k_r >_{\mathrm{rel}}$ the corresponding relative, average radiative rate constant.

For MeOthaU in Tris buffer, the fractional non-emitting population (at an excitation wavelength of 390 nm) was calculated from the ratio of the quantum yields in buffer and dioxane, 0.01 and 0.51 (at excitation wavelength 360 nm), respectively, and the ratio of the corresponding average lifetimes, 0.17 ns and 3.0 ns, respectively, assuming that $< k_r >$ depends only on the square of the refractive index of the solvent (refractive indices of 1.350 and 1.420, for Tris and dioxane, respectively). The latter assumption is supported by the similarity of the ε_{max} values in the two solvents, 19770 M^{-1} cm^{-1} and 20800 M^{-1} cm^{-1}, respectively.
This gave a fractional non-emitting population of 62%.

For the MeOthaU -containing oligos, there is a reduction in the value of ε_{max} compared with that of MeOthaU in dioxane. This was taken as an indication of a reduction in $< k_r >$ in the oligo and the ratio of ε_{max} values (see below) was included in the value of $< k_r >_{rel}$ in eq. 6.

ε_{max} (AXA SS)/ ε_{max}(dioxane) = 0.85

ε_{max} (AXT SS)/ ε_{max}(dioxane) = 0.86

ε_{max} (AXA DS)/ ε_{max}(dioxane) = 0.63

ε_{max} (AXT DS)/ ε_{max}(dioxane) = 0.58

S3 Calculation of species-specific quantum yields

Quantum yields of the individual emitting species in the oligonucleotides were calculated according to eq. 7.

$$\emptyset_i = \frac{\tau_i}{\langle\tau\rangle_{\text{diox}}} < k_r >_{rel} \emptyset_{\text{diox}} \quad (7)$$

where $\emptyset_i$ is the quantum yield of the *i*th emitting species in the oligo, τ_i is the lifetime of *i*th emitting species, $\langle\tau\rangle_{\text{diox}}$ is the number-average lifetime of the nucleoside in dioxane (3.0 ns), $< k_r >_{rel}$ is the average radiative rate constant for the oligo relative to that of $MeO^{th}aU$ in dioxane (see S2 above), and $\emptyset_{\text{diox}}$ is the quantum yield of $MeO^{th}aU$ in dioxane (0.51 at the excitation wavelength used to measure $\langle\tau\rangle_{\text{diox}}$).
This assumes that there is negligible difference between the radiative rate constants of the different emitting $MeO^{th}aU$ species. This assumption is supported by the similarity in the values of ε_{max} for $MeO^{th}aU$ in dioxane and Tris, in spite of a large difference in the composition of the excited state populations in the two solvents.

S4 Supplementary Tables

Table S1 Two-photon brightness ($\phi\sigma_2$) and two-photon cross-sections (σ_2) of extended 6-aza-uridines in dioxane.

Substituent	$\phi\sigma_2$ / GM	σ_2/GM (wavelength)
F	1.6	5.1 (750 nm)
Me	2.4	6.3 (770 nm)
OMe	10	13 (780 nm)
OH	10	14 (780 nm)
NMe_2	18	90 (840 nm)

Table S2 Fractional amplitudes (A-factors) A_i, fractional contributions to steady-state intensity, S_i and average lifetimes (<τ>), as a function of emission wavelength for $MeO^{th}aU$ nucleoside in dioxane, excited at 360 nm. Globally fitted lifetimes: τ_1 = 0.30 ns and τ_2= 4.2 ns. The quantum yield at this excitation wavelength is 0.51.

λ_{em} / nm	A_1	A_2	S_1	S_2	<τ> /ns
**470**	0.37	0.63	0.04	0.96	2.8
**490**	0.32	0.68	0.03	0.97	3.0
**510**	0.28	0.73	0.03	0.97	3.1

Table S3 Fractional amplitudes (A-factors) A_i, fractional contributions to steady-state intensity, S_i and average lifetimes (<τ>), as a function of emission wavelength for MeOthaU nucleoside in Tris, excited at 390 nm. Globally fitted lifetimes: τ_1 = 0.11 ns, τ_2= 0.18 ns, τ_3= 0.47 ns

λ_{em} / nm	A_1	A_2	A_3	S_1	S_2	S_3	<τ> /ns
**505**	0.28	0.70	0.02	0.18	0.76	0.06	0.17
**525**	0.18	0.80	0.02	0.12	0.83	0.05	0.17
**545**	0.11	0.88	0.01	0.07	0.90	0.03	0.18

Table S4 Two-photon brightness ($\sigma_2\phi$) and two-photon cross-section (σ_2), as a function of excitation wavelength, for the oligos as single and double strands. Cross-section values were calculated using quantum yields measured at an excitation wavelength of 394 nm.

Sample	λ_{ex} / nm	$\phi\sigma_2$ /GM	σ_2 /GM
SS AXA	760	0.61	12
	770	0.65	13
	780	0.66	13
	790	0.61	12
	800	0.61	12
DS AXA	760	0.26	6.4
	770	0.24	6.1
	780	0.31	7.8
	790	0.20	5.1
	800	0.26	6.6
SS AXT	760	0.70	10
	770	0.78	11
	780	0.82	11
	790	0.70	10
	800	0.67	9.5
DS AXT	760	0.21	5.3
	770	0.20	4.9
	780	0.25	6.2
	790	0.17	4.3
	800	0.23	5.8

Supplementary Figures

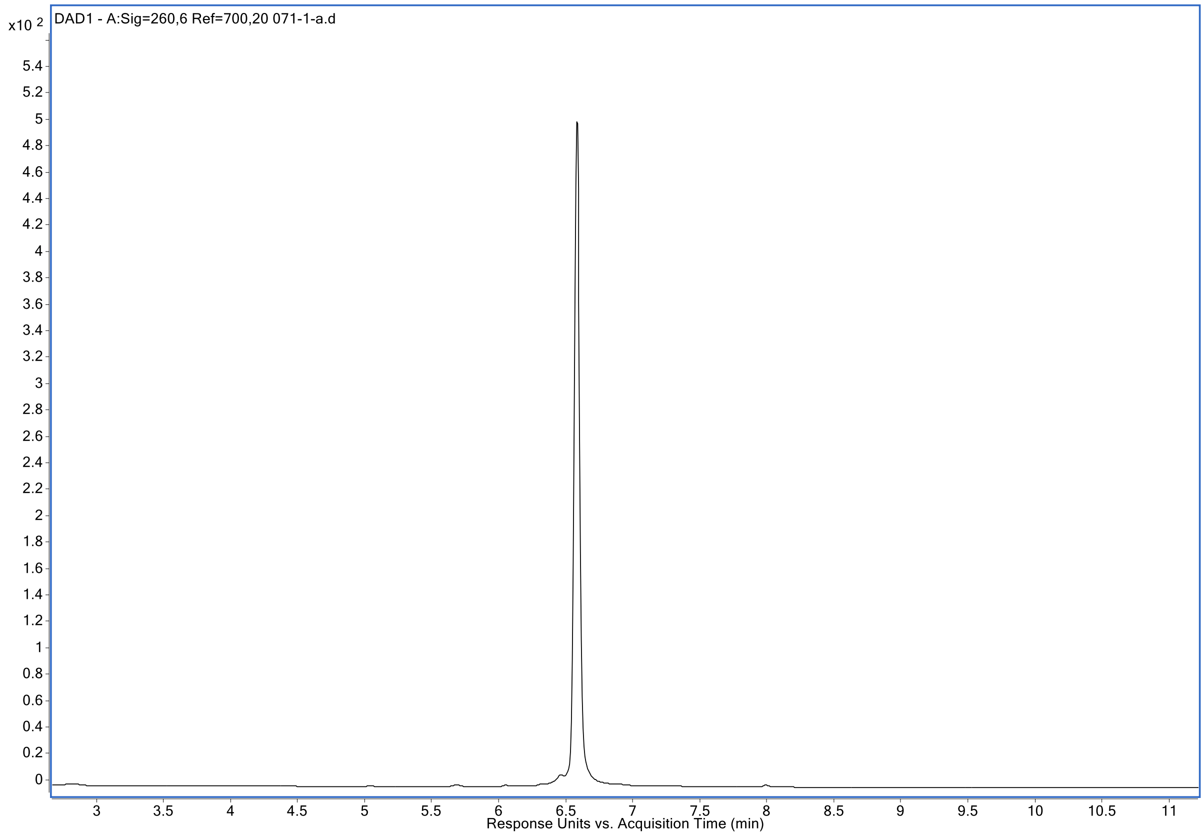

Figure S1 LC of AXA with peaks detected at 260 nm.

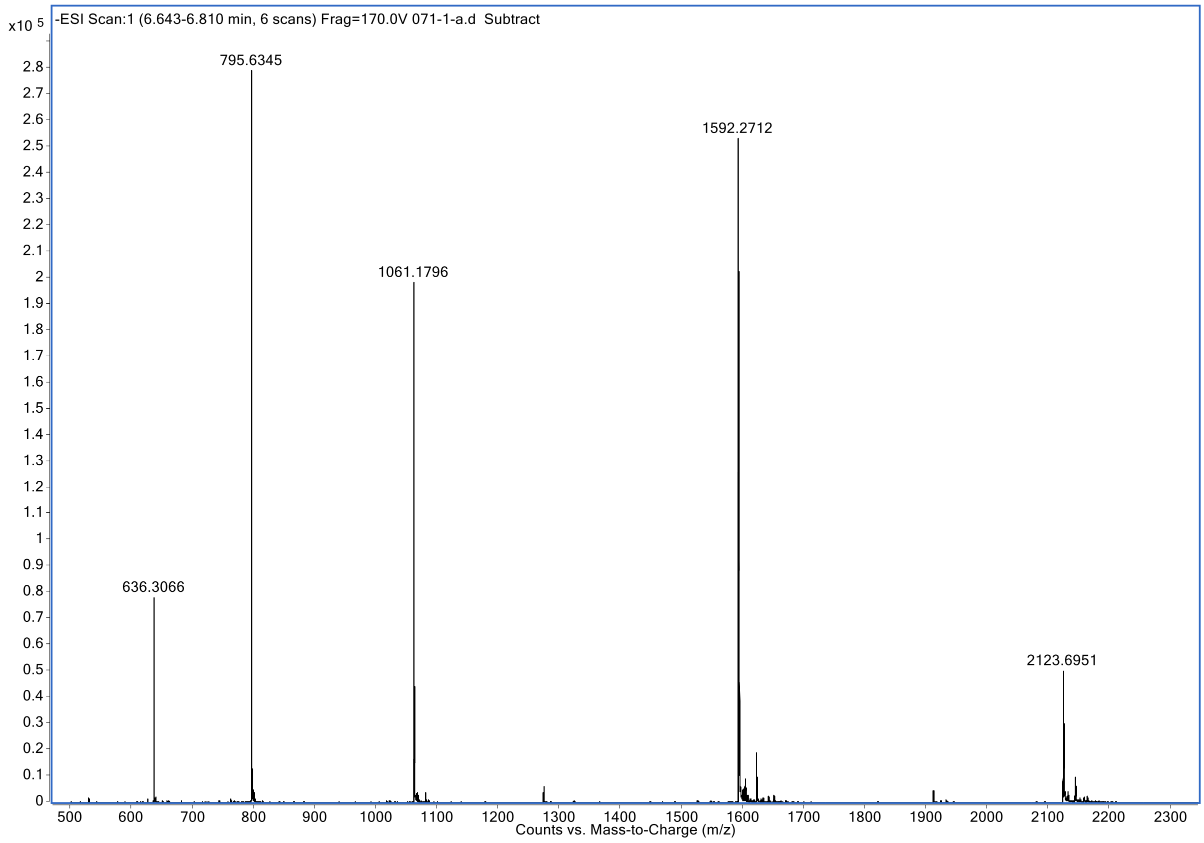

Figure S2 ESI-TOFMS of AXA

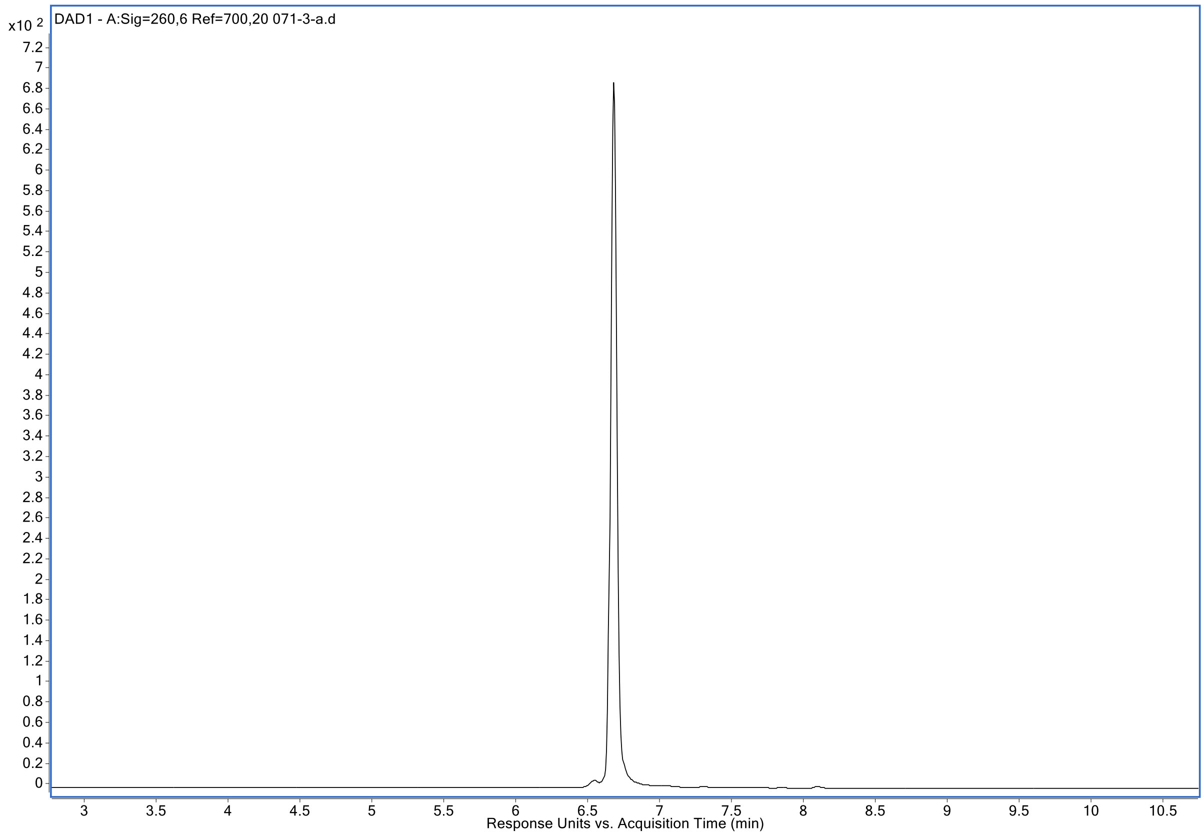

Figure S3 LC of AXT with peaks detected at 260 nm.

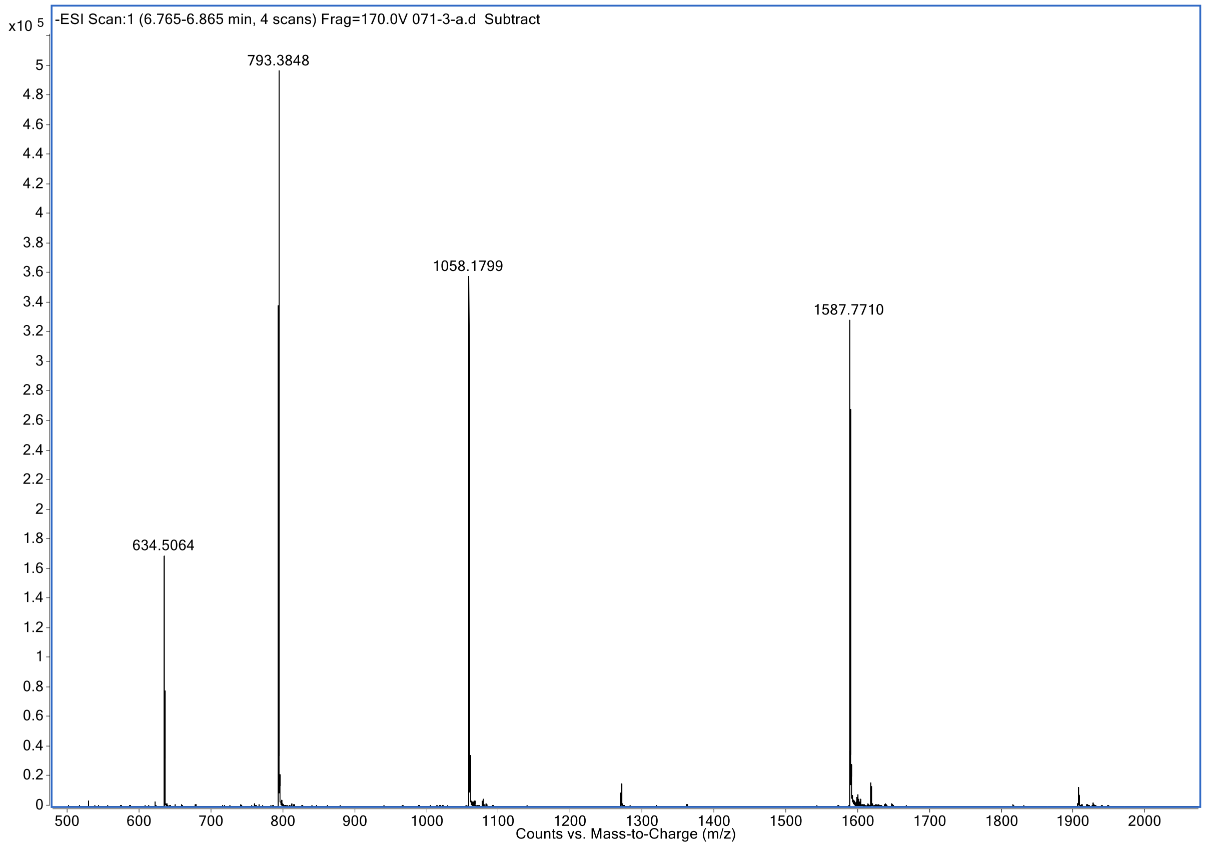

Figure S4 ESI-TOFMS of AXT

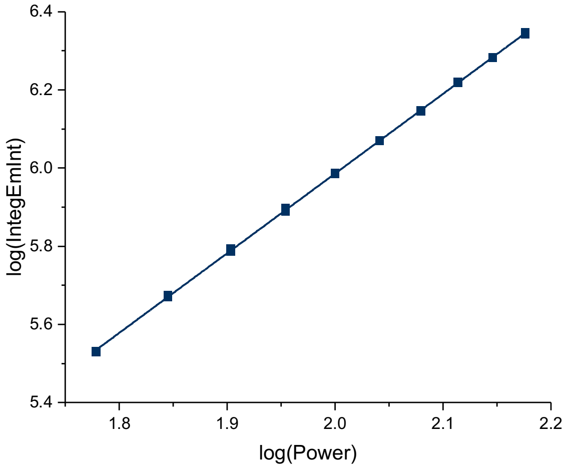

Figure S5 Log-log plot of emission intensity versus laser power for 780-nm excitation of MeOthaU nucleoside in dioxane. Gradient is 2.0.

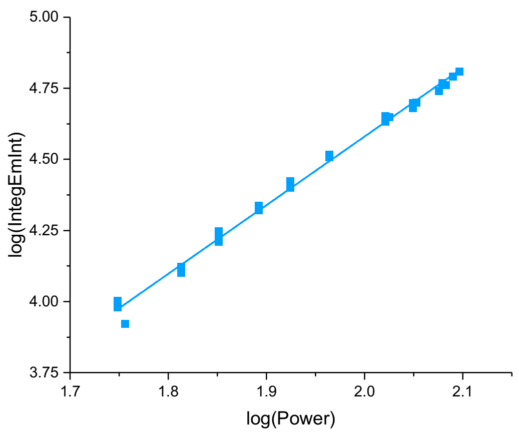

Figure S6 Log-log plot of emission intensity versus laser power for 780-nm excitation of MeOthaU nucleoside in Tris. Gradient is 2.4.

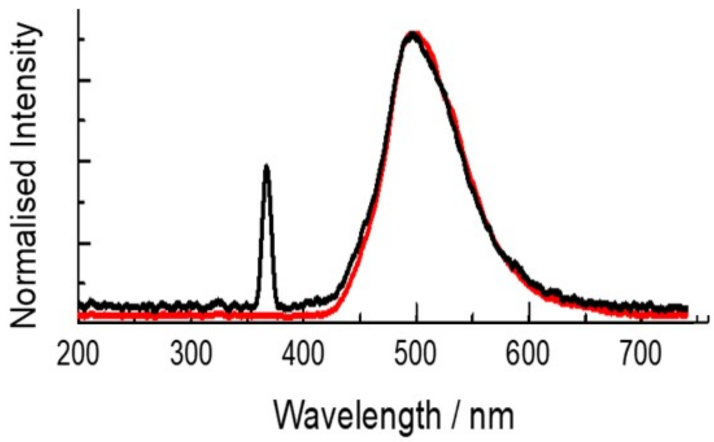

Figure S7 Comparison of the emission spectra of the MeOthaU nucleoside in dioxane under one-photon excitation at 370 nm (black) and two-photon excitation at 780 nm (red).

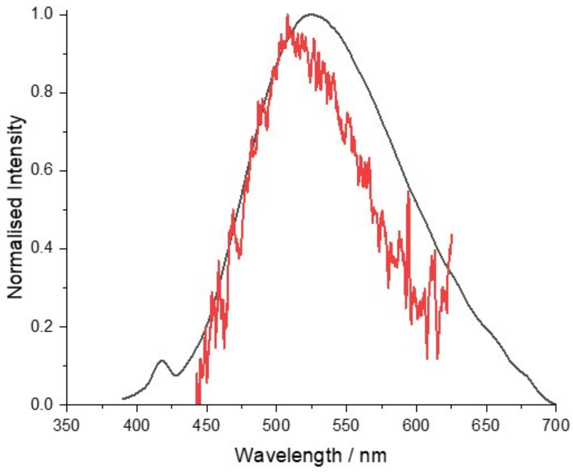

Figure S8 Comparison of the emission spectra of the MeOthaU nucleoside in Tris buffer under one-photon excitation at 390 nm (black) and two-photon excitation at 780 nm (red).

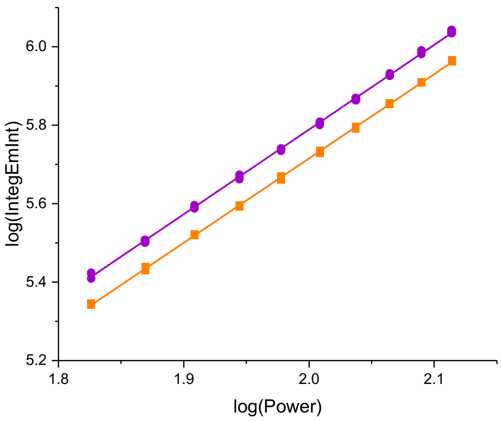

Figure S9 Log-log plot of emission intensity versus laser power for 780-nm excitation of single-strand oligos AXA (orange) and AXT (purple). Gradients are 2.1 and 2.0, respectively.

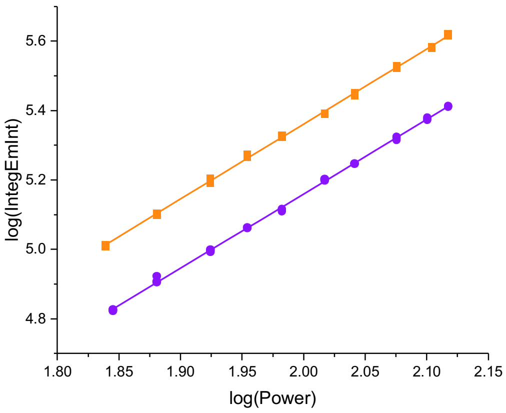

Figure S10 Log-log plot of emission intensity versus laser power for 780-nm excitation of double-strand oligos AXA (orange) and AXT (purple). Gradients are 2.2 and 2.1, respectively.

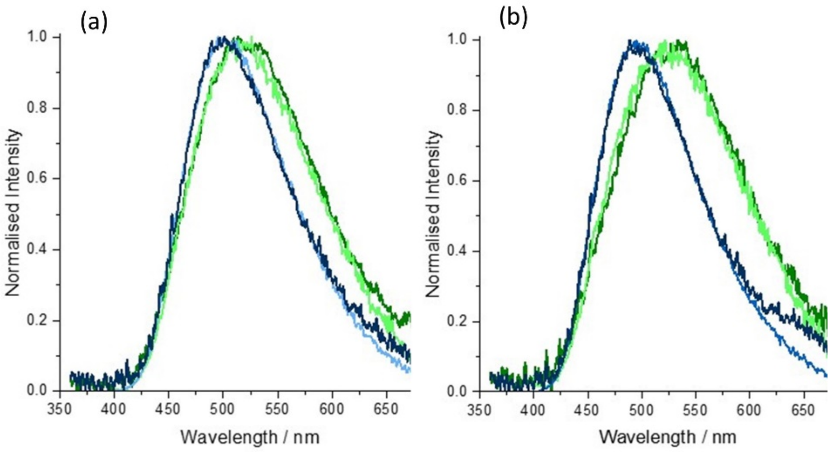

Figure S11 Comparison of the emission spectra under one-photon excitation, at 375 nm, and two-photon excitation, at 780 nm, for oligonucleotides as single and double strands. (a) Oligonucleotide AXA. (b) Oligonucleotide AXT. Single strand under 1P-excitation (pale blue) and 2P-excitation (dark blue); double strand under 1P-excitation (pale green) and 2P-excitation (dark green).

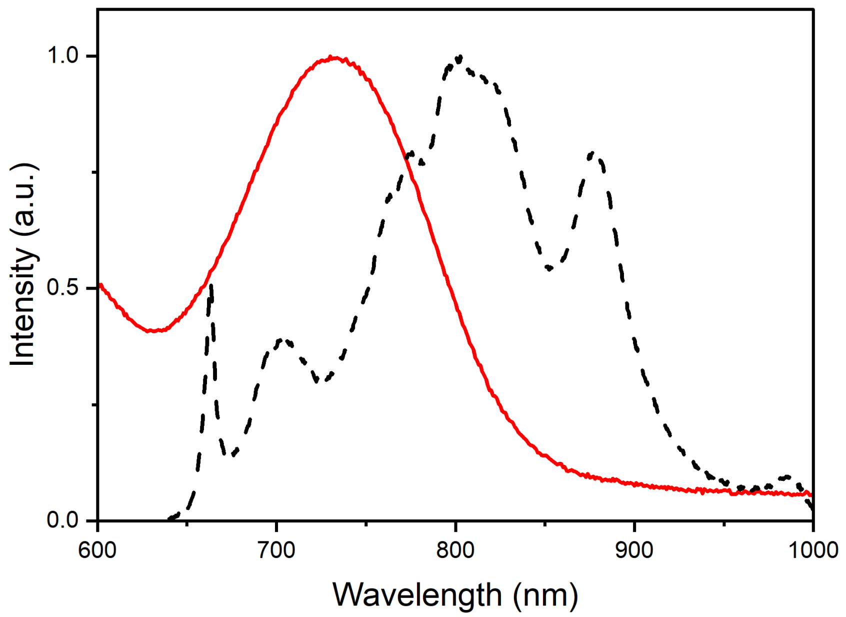

Figure S12 Normalised absorption of 900 nM MeOthaU nucleoside in Tris buffer plotted at twice the wavelength (red) showing the overlap with the broadband Ti:S laser spectrum (black dashed).

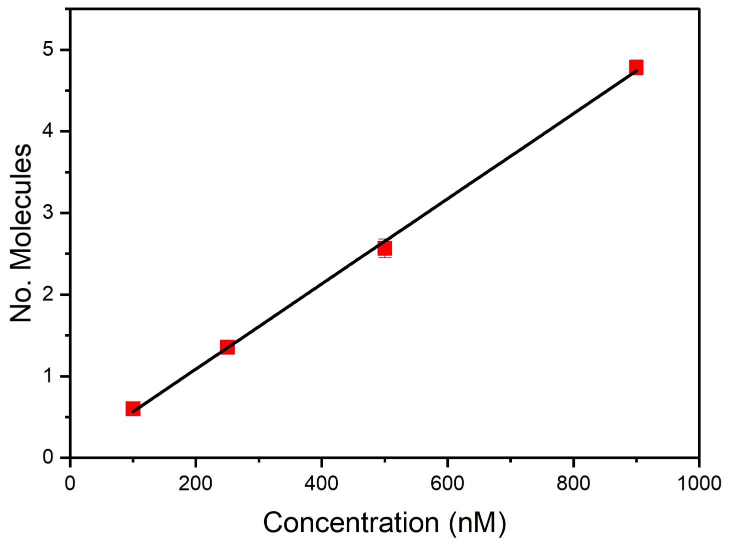

Figure S13 Dilution series from a known concentration of MeOthaU nucleoside in Tris showing a linear relationship between the concentration and the number of molecules detected by 2P microscopy; the y axis intercept is 0.04 ± 0.06. The excitation power was 9.6 mW.

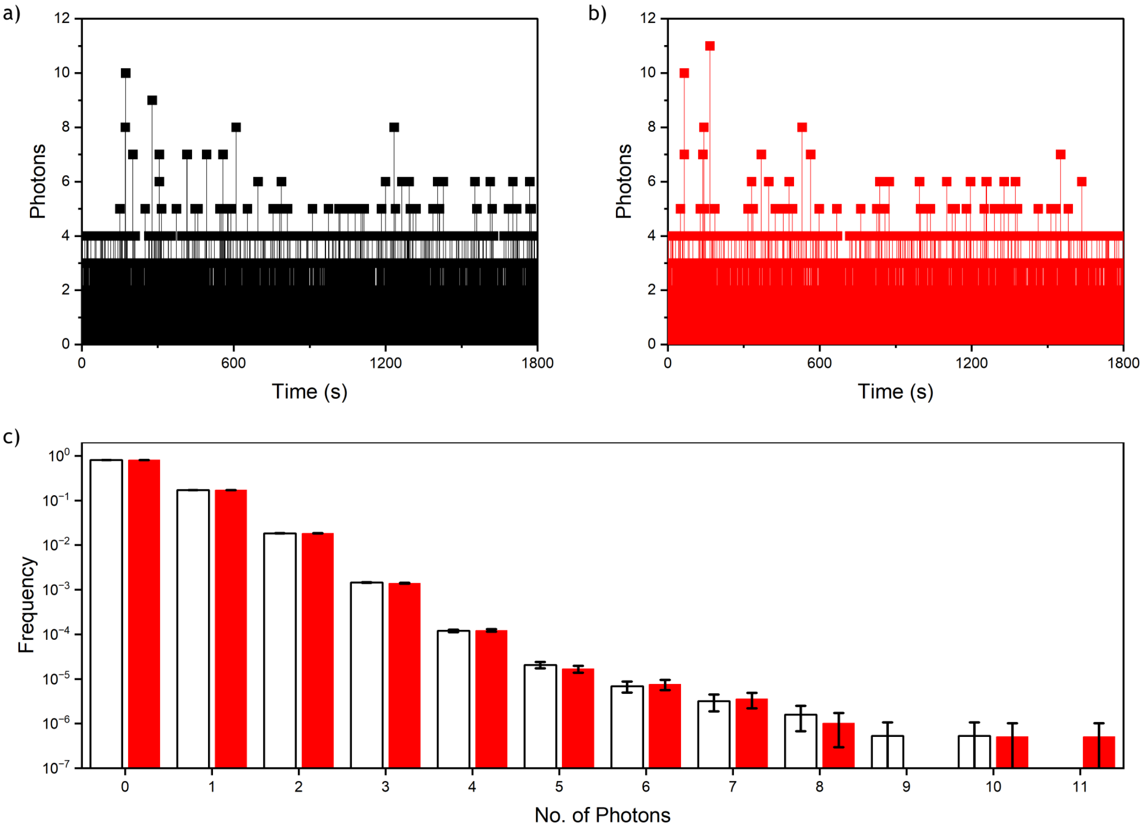

Figure S14 MCS traces of a) buffer and b) 100 pM MeOthaU nucleoside following 2P FCS. The excitation power was 9.0 mW using a custom spectrum cropping the components above 850 nm. The traces are from three separate 10 minute measurements that are appended together to make one 30 minute trace. The bin width is 1 ms. Photons that arrived less than 1 ns after the laser pulse were discarded. c) A photon counting histogram of the data in a) and b) with the buffer in black and MeOthaU in red.

References

1. P. A. Hopkins, R. W. Sinkeldam and Y. Tor, Visibly Emissive and Responsive Extended 6-Aza-Uridines, *Org. Lett.*, 2014, **16**, 5290-5293.
2. D. Nobis, H. G. Sansom and S. W. Magennis, Pulse-shaped broadband multiphoton excitation for single-molecule fluorescence detection in the far field, *Methods Appl. Fluoresc.*, 2023, **11**, 017001.
3. V. V. Lozovoy, I. Pastirk and M. Dantus, Multiphoton intrapulse interference. IV. Ultrashort laser pulse spectral phase characterization and compensation, *Opt. Lett.*, 2004, **29**, 775-777.
